# Combined Anterior Cruciate Ligament and Posterolateral Corner Reconstruction With a Single Femoral Tunnel Using Rectus Femoris and Semitendinosus Autografts

**DOI:** 10.1002/atn2.70275

**Published:** 2026-09-25

**Authors:** Pedro Henrique de Oliveira Paolucci, Riccardo Gomes Gobbi, Flavio de Fava Sanches, Said El Orra, Rodrigo Schuwarten Aidar

**Affiliations:** ^1^ Grupo de Joelho, Instituto de Ortopedia e Traumatologia, Hospital das Clínicas HCFMUSP Faculdade de Medicina da Universidade de São Paulo São Paulo Brazil

## Abstract

We describe a reproducible, anatomically based technique for combined anterior cruciate ligament (ACL) and posterolateral corner reconstruction using a single femoral tunnel and 2 autografts: rectus femoris and semitendinosus. Part of the rectus femoris and semitendinosus grafts is prepared for anatomic ACL reconstruction, and a shared femoral tunnel accommodates the ACL femoral socket while providing a lateral exit for proximal posterolateral corner fixation. The remaining rectus femoris graft exiting the femoral tunnel is used to reconstruct the popliteus tendon, and the remaining semitendinosus graft is used to reconstruct the fibular collateral ligament and popliteofibular ligament. This approach enables combined ACL and posterolateral corner reconstruction through a single femoral tunnel, preserving anatomic principles and minimizing the risk of tunnel convergence.

## Graphical abstract

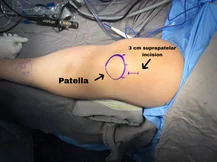

**VIDEO 1:** Technique for combined anterior cruciate ligament and posterolateral corner reconstruction with a single femoral tunnel using rectus femoris and semitendinosus autografts. The procedure is performed on the left knee with the patient in the supine position. The video illustrates rectus femoris and semitendinosus graft harvest and preparation, creation of a shared femoral tunnel for anterior cruciate ligament and posterolateral corner reconstruction, graft passage and fixation, and final construct assessment. The key principle of the technique is the use of a single femoral tunnel to achieve anatomic reconstruction of the anterior cruciate ligament and posterolateral corner while minimizing femoral tunnel convergence and preserving bone stock. Video content can be viewed at https://doi.org/10.1002/atn2.70275.

The fibular collateral ligament (FCL) and popliteofibular ligament (PFL) complex play essential roles in controlling varus and external rotation of the knee. Detailed knowledge of the posterolateral corner (PLC) anatomy is fundamental for reliable reconstruction techniques, as shown in classic morphological analyses.^1^ In vivo studies have shown that sectioning of these structures produces predictable instability patterns, reinforcing the importance of anatomic reconstruction to restore knee stability.^2^ Consistent with these findings, recent reviews report higher failure rates in nonanatomic PLC reconstructions compared with anatomic techniques, supporting current surgical principles.^3^

In multiligament knee injuries, femoral tunnel convergence remains a significant concern. Single femoral tunnel strategies have therefore been proposed to preserve bone stock while maintaining anatomic reconstruction.^4^ Proper tunnel orientation and drilling angles are critical to ensure safe trajectories and adequate bone bridges.^5^ Additionally, reproducible landmarks, such as the posterior cartilage margin of the lateral femoral condyle, improve accuracy in femoral tunnel placement in complex cases.^6^

Regarding graft selection, the semitendinosus (ST) is widely used in anatomic PLC reconstruction.^7^, ^8^, ^9^, ^10^ For anterior cruciate ligament (ACL) reconstruction, the rectus femoris (RF) has gained attention because of its predictable morphology, facilitating combined procedures without the need for allografts. When necessary, the peroneus longus tendon provides comparable outcomes and serves as an alternative graft option.^10^, ^11^ The purpose of this technical note is to describe a combined ACL and PLC reconstruction technique using RF and ST autografts through a single femoral tunnel, aiming to achieve anatomic restoration while minimizing tunnel convergence.

## SURGICAL TECHNIQUE

The key steps are summarized in Table 1 and shown in Video 1.

**TABLE 1:** Stepwise Surgical Technique for Combined ACL and PLC Reconstruction Using a Single Femoral Tunnel Using Rectus Femoris and Semitendinosus Autografts

**Stage**	**Principal Step**	**Knee Position/Maneuvers**	**Fixation/Implants**	**Key Notes**
Examination under anesthesia	Anterior drawer, Lachman, varus/valgus stress, and dial test	Supine position with a lateral post for applying valgus stress and a foot support	‐	Determination of injury severity and associated instabilities
Graft preparation	‐ RF (double‐strand) + ST (single‐strand) → ACL ‐ RF (single‐strand) → popliteus tendon (PT) ‐ ST (single‐strand) → FCL and PFL	Knee positioned at 20° of flexion	Harvesting of the RF and ST tendons using a stripper, and whipstitch both with 1.0 Vicryl	Check the grafts’ length and diameter before drilling
Tunnels	‐ Single femoral tunnel for the ACL and PLC ‐ Anatomic tibial tunnel for the ACL ‐ Fibular tunnel for the FCL and PFL ‐ Tibial tunnel for the FCL and PFL	‐ Femoral and tibial tunnels (ACL) under arthroscopic visualization ‐ Fibular and tibial tunnels (PLC) under fluoroscopic visualization	‐ Initial 8‐mm reamer for the femoral and tibial tunnels (ACL) ‐ Five‐millimeter reamer for the fibular tunnel ‐ Seven‐millimeter reamer for the tibial tunnel (PLC)	Protection of the peroneal nerve which is located posteromedial to the biceps
Graft passage	‐ ACL graft: femoral → tibial tunnel ‐ PLC graft: femoral → fibular → tibial tunnel	‐ Fixation of the ACL graft first then the PLC ‐ FCL fixated with 30° of flexion, neutral rotation, and slight valgus ‐ PT and PFL fixated with 60° flexion, neutral rotation, and slight valgus	‐ Graft passage using a passing suture with loop ‐ Fixation with interference screw	‐ Adequate tensioning of the grafts ‐ Ensure the restoration of normal varus and external rotation

ACL, anterior cruciate ligament; FCL, fibular collateral ligament; PFL, popliteofibular ligament; PLC, posterolateral corner; PT, popliteus tendon; RF, rectus femoris; ST, semitendinosus.

### Patient Positioning and Preoperative Examination

The patient is placed supine with a proximal thigh tourniquet. A valgus stress post and foot support are positioned. After spinal anesthesia, the knee is examined to confirm ACL and PLC injuries.

### Graft Harvesting and Preparation

**FIGURE 1:**
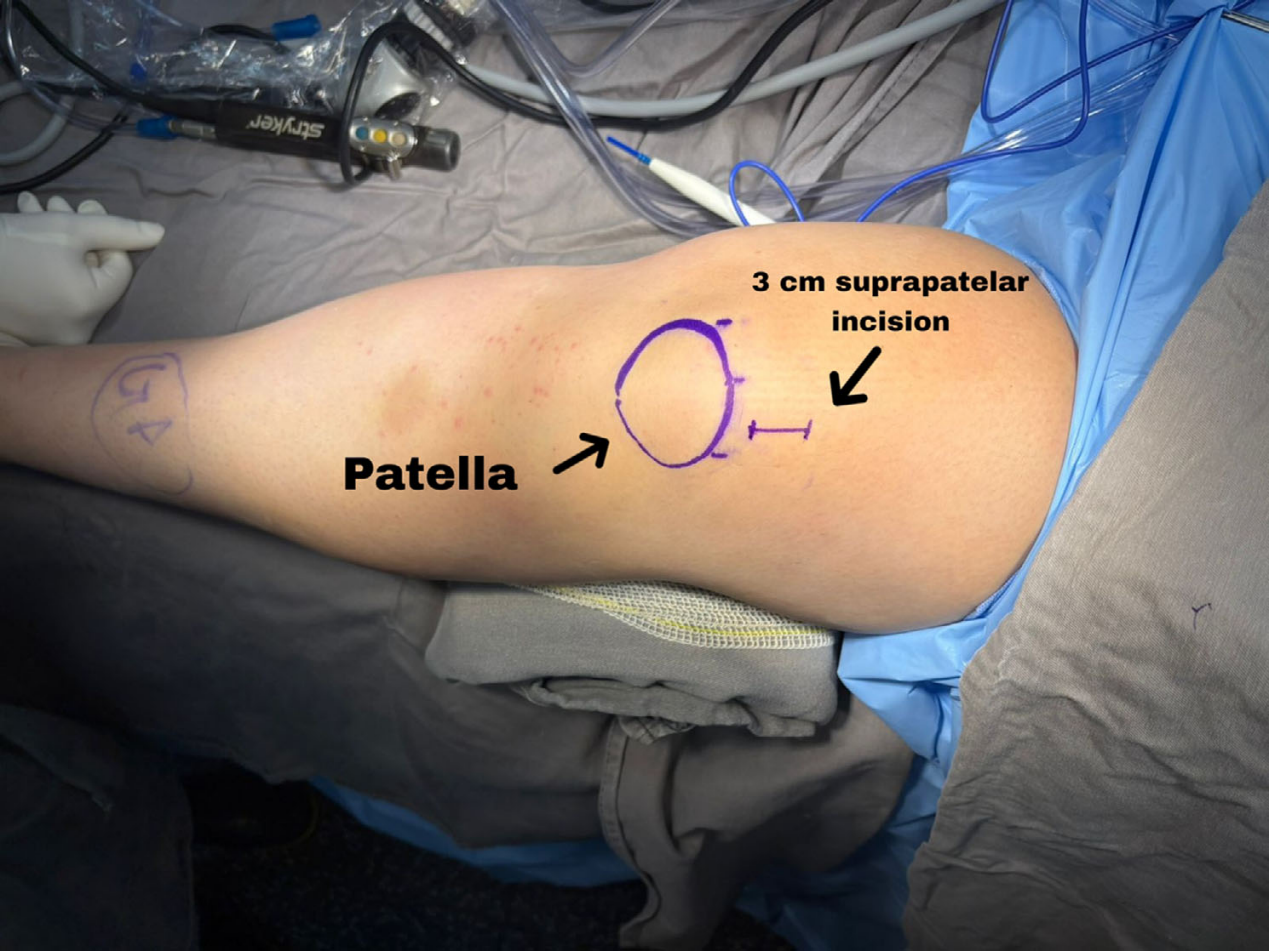
Anterior view of the left knee. A 3‐cm longitudinal incision is made in the suprapatellar region between the middle and lateral thirds of the patella.

**FIGURE 2:**
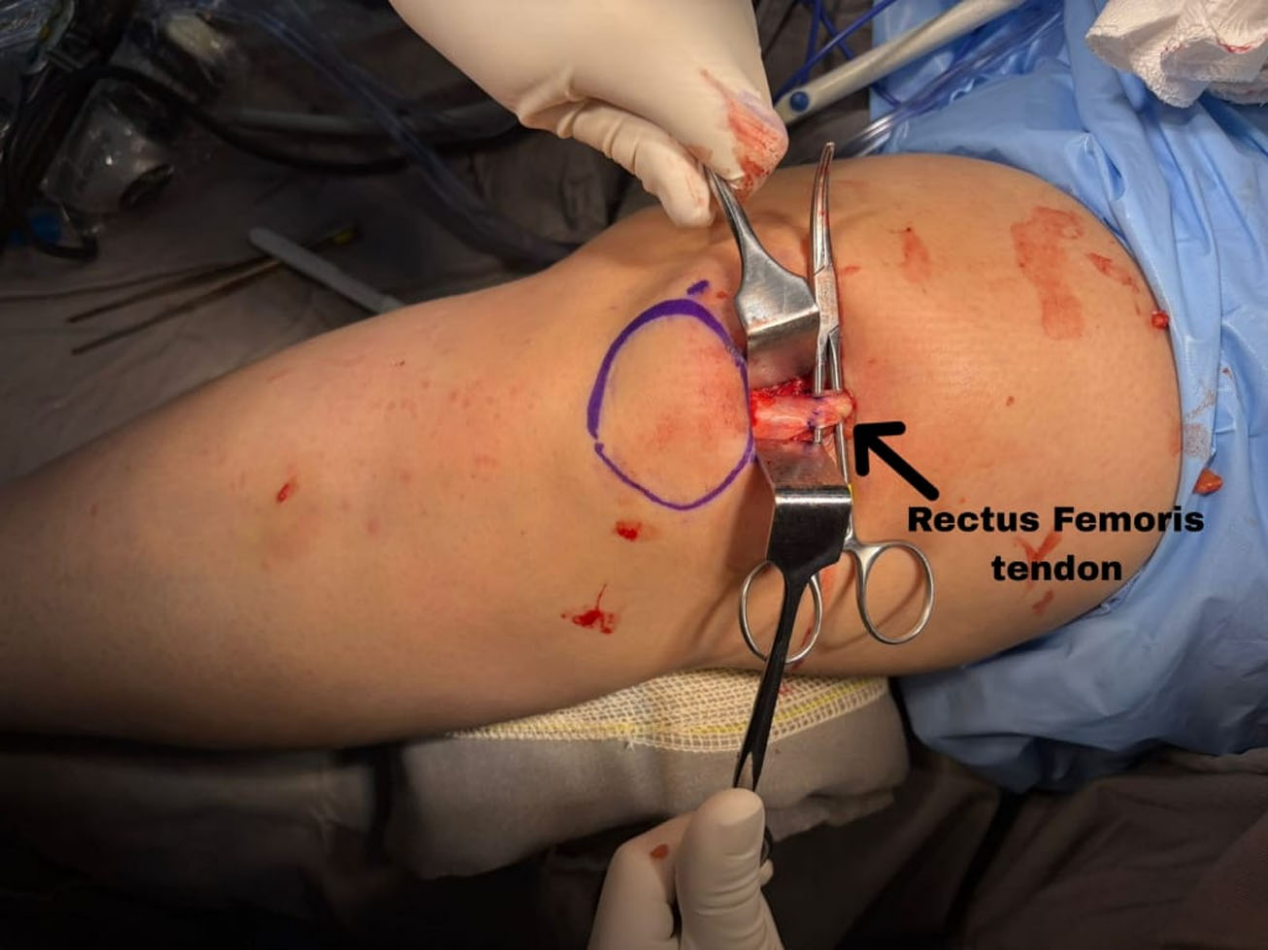
Anterior view of the left knee following skin incision and soft tissue dissection. The rectus femoris tendon is identified and isolated with a Kelly forceps.

For the RF, a 3‐cm longitudinal incision is made from the suprapatellar region between middle and lateral thirds, extending proximally. Two parallel longitudinal incisions, approximately 3 mm apart and starting 2 cm proximal to the patella, are made to define a tendon strip approximately 10 mm in width. The tendon is detached, sutured with Vicryl 1‐0 (Ethicon). The proximal portion of the tendon is then carefully separated from the vastus intermedius and released proximally with a tendon stripper at 20° knee flexion (Figures 1 and 2).

For the ST, a 3‐cm longitudinal incision is made between the tibial tubercle and medial tibial border. The tendon is identified, detached, and released proximally with a tendon stripper.

**FIGURE 3:**
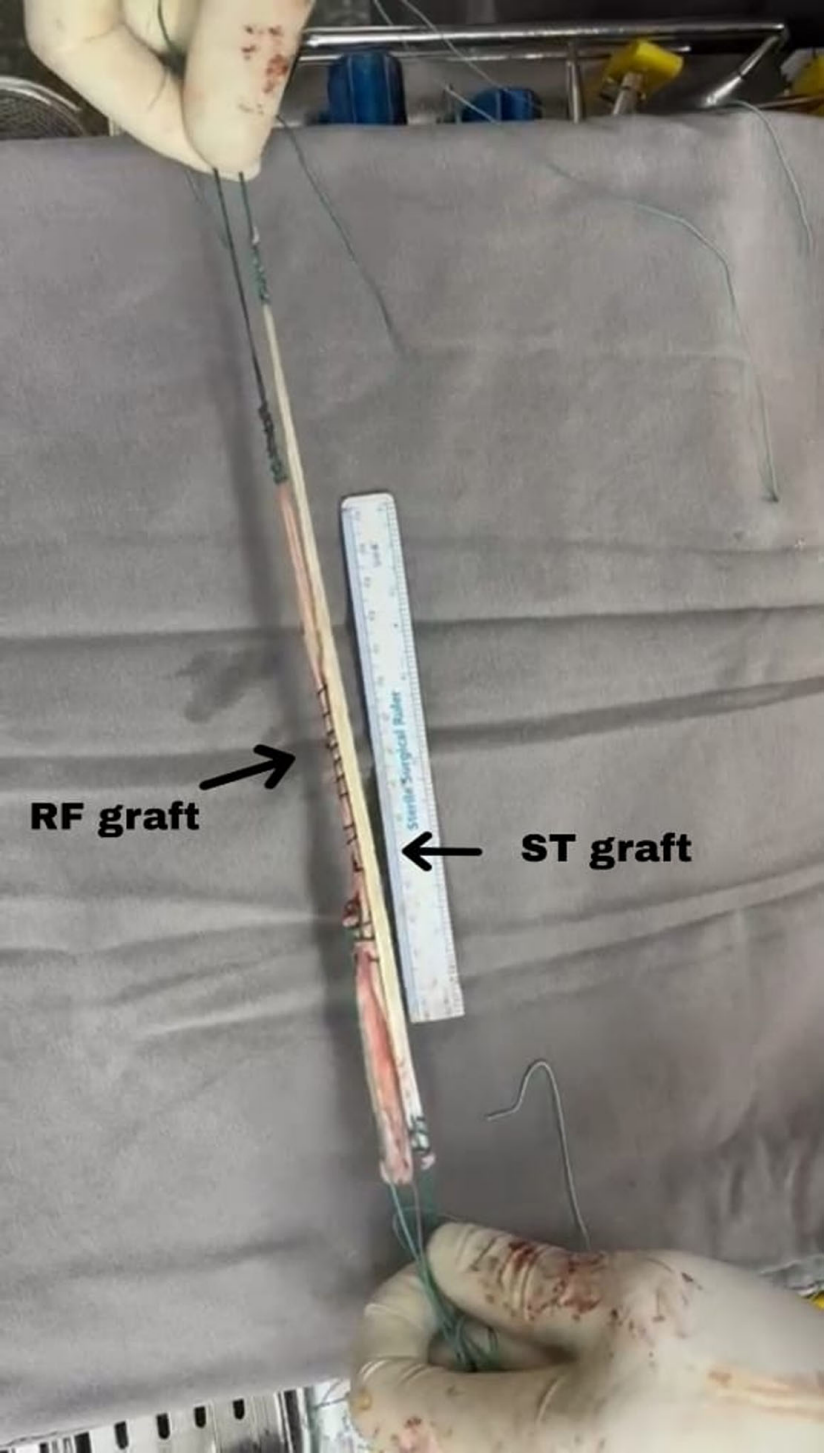
RF and ST grafts after initial preparation. RF is prepared double‐stranded with a single‐stranded tail; ST is single‐stranded. (RF, rectus femoris; ST, semitendinosus.)

**FIGURE 4:**
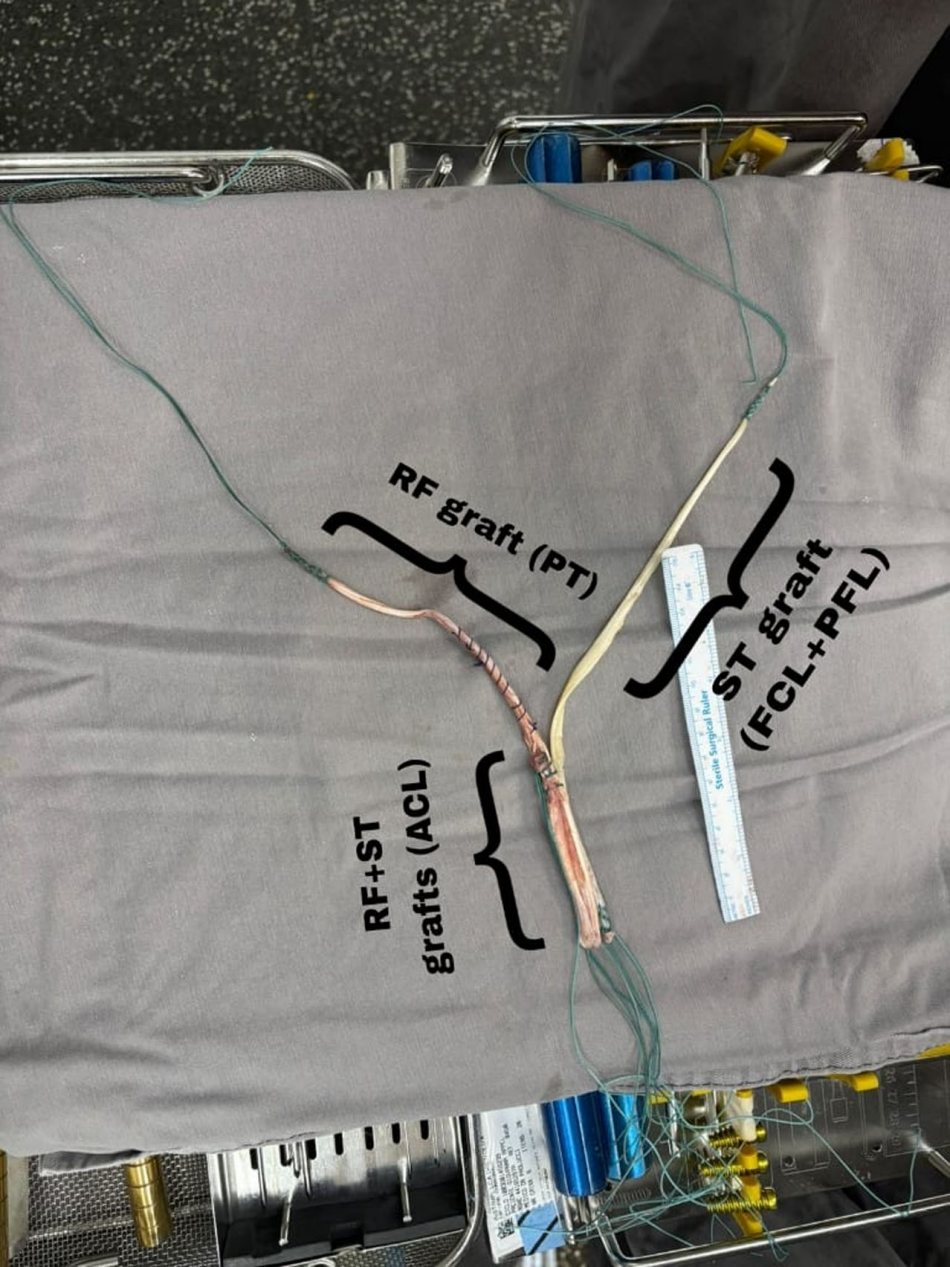
RF and ST grafts after final preparation. The double‐stranded RF and a part of ST form the ACL; the remaining RF serves as PT, and the remaining ST forms FCL and PFL. (ACL, anterior cruciate ligament; FCL, fibular collateral ligament; PFL, popliteofibular ligament; PT, popliteus tendon; RF, rectus femoris; ST, semitendinosus.)

RF is prepared double‐stranded with a single‐stranded tail; ST is single‐stranded (Figure 3). The double‐stranded RF and part of ST form the ACL ligament; the remaining RF serves as popliteus tendon (PT), and the remaining ST forms FCL and PFL ligaments (Figure 4).

### Surgical Approach

**FIGURE 5:**
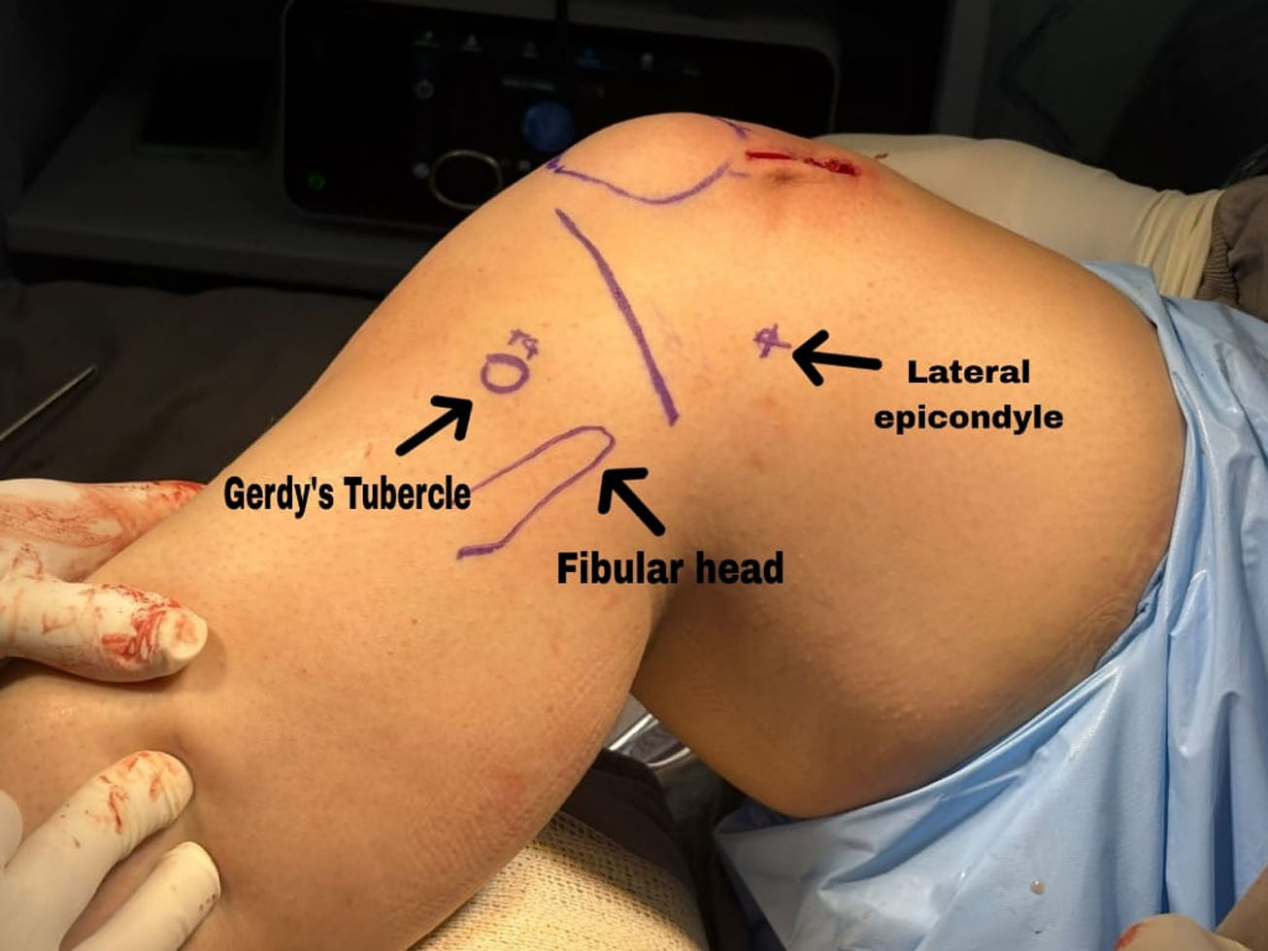
Anterolateral view of the left knee in 90° flexion, illustrating the principal anatomical landmarks: Gerdy's tubercle, lateral epicondyle, and the fibular head. The “hockey stick” incision is made proximal to the lateral epicondyle and extends distally between the fibular head and Gerdy's tubercle.

**FIGURE 6:**
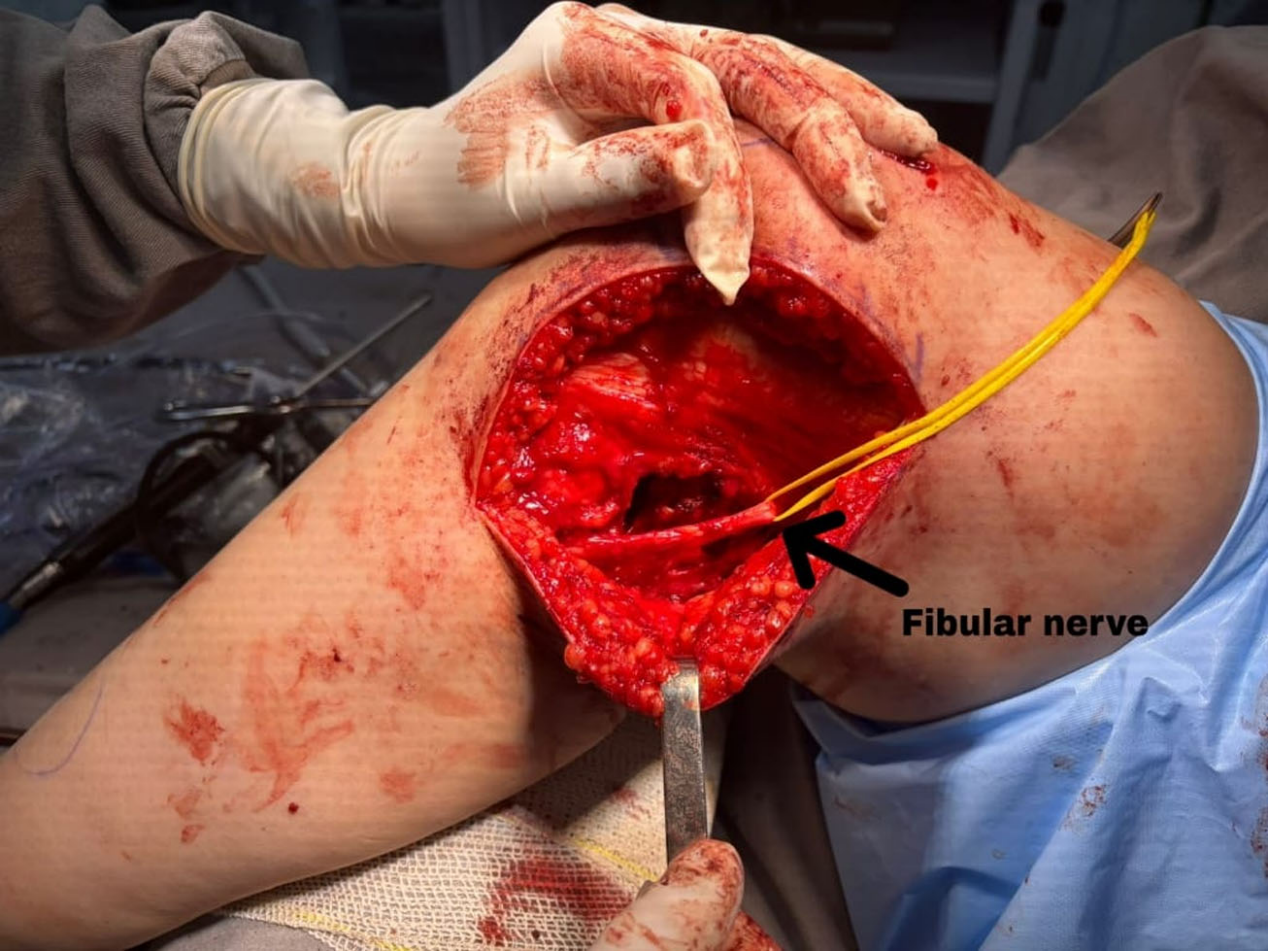
Anterolateral view of the left knee in 90° flexion after incision and dissection. The fibular nerve is identified and protected with a vessel loop.

Diagnostic arthroscopy is carried to rule out additional lesions. A hockey stick incision is made from the lateral epicondyle extending distally between the fibular head and Gerdy's tubercle (Figure 5). Posterior skin flap is raised; fibular nerve is identified and protected (Figure 6). Iliotibial band incision is performed to visualize the FCL and PT origins. Blunt dissection to fibular head and posterolateral tibia was done to facilitate tunnel preparation. Tibiofibular instability was noted intraoperatively and fixed with a 3.5‐mm cancellous screw (IOL Implantes Ortopédicos, São Paulo, SP, Brazil).

### Tunnel Preparation

**FIGURE 7:**
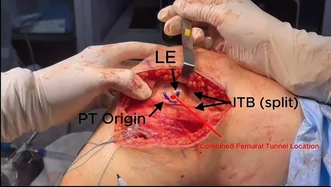
Lateral view of the left knee. The ITB is incised and the origins of FCL and PT. The common femoral tunnel is located at a midpoint between the origins of the FCL and PT. (FCL, fibular collateral ligament; ITB, iliotibial band; LE, lateral epicondyle; PT, popliteus tendon.)

ACL tunnels are created using the standard outside‐in technique under arthroscopy. A common femoral tunnel serves both the ACL and PLC. A midpoint between the FCL and PT origins is identified as a reference (Figure 7). A guide pin is passed through this point to exit at the native ACL footprint. The tibial tunnel is created conventionally: a guide pin is passed medial to the tibial tubercle through the ST graft incision and exits at the ACL tibial footprint. An 8‐mm drill‐bit is used to create both tunnels. A suture loop is placed through the femoral tunnel to aid graft passage. For the fibular tunnel, a guide pin is aimed from the fibular head‐neck junction, anterolateral to posteromedial, and a 5‐mm tunnel is drilled and a passing suture is placed. For the PLC tibial tunnel, a guide pin is passed anterior to posterior with a retractor placed on the posterolateral tibial ridge, aiming medial and distal to the Gerdy's tubercle, with the exit 1 cm medial and 1 cm proximal to the fibular tubercle. A 7‐mm tunnel is drilled, and a passing suture is placed. Tunnel positions are confirmed under fluoroscopy.

### Graft Passage

**FIGURE 8:**
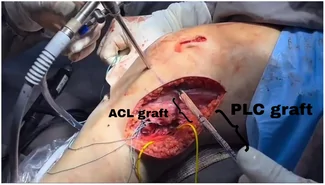
Lateral view of the left knee showing the anterograde passage of the ACL graft from the femoral tunnel to the tibial tunnel, with the remaining graft preserved for subsequent PLC reconstruction. (ACL, anterior cruciate ligament; PLC, posterolateral corner.)

**FIGURE 9:**
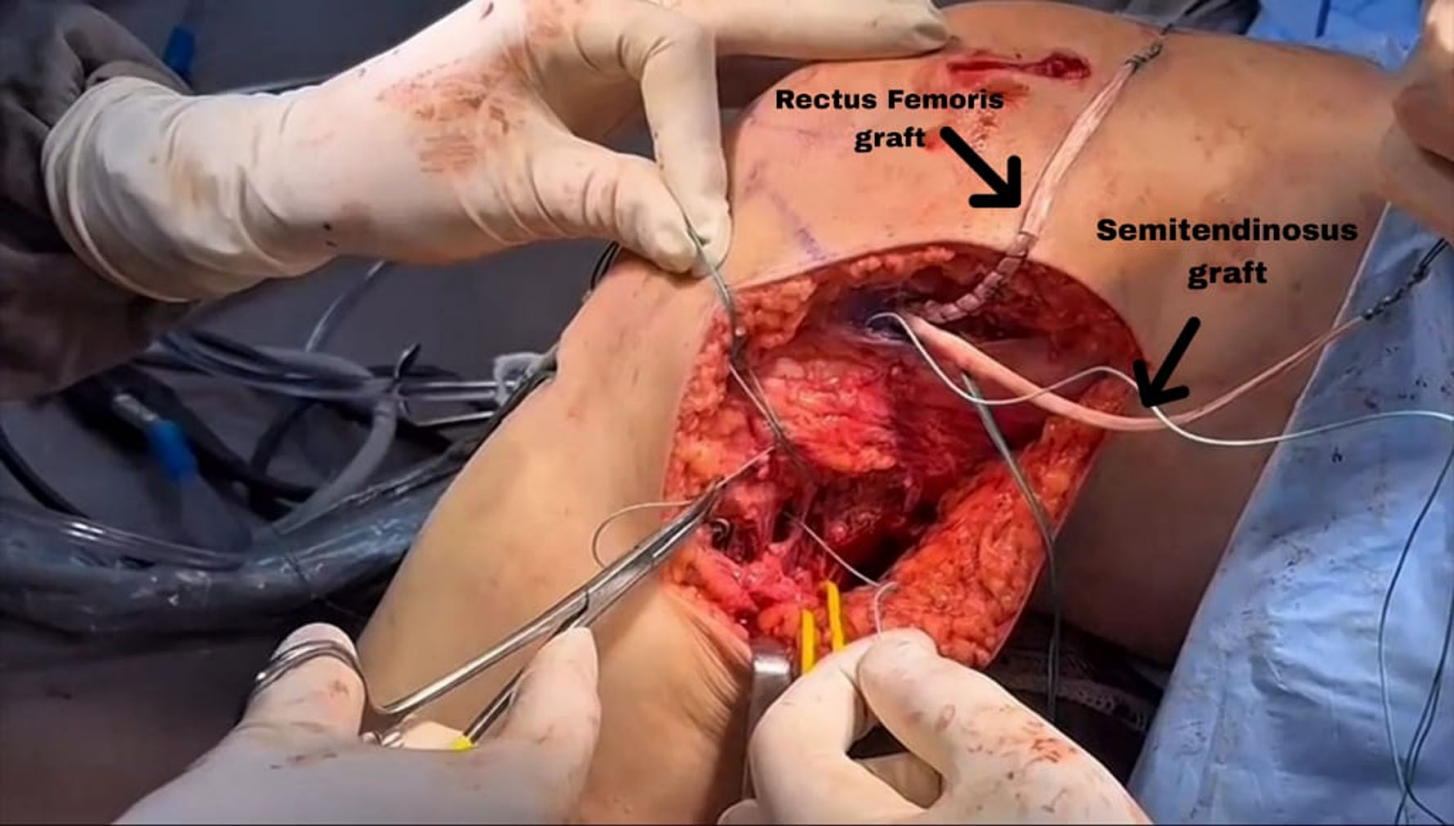
Lateral view of the left knee after the passage of the ACL graft. The remaining grafts which are composed of the single‐stranded RF and ST grafts are then passed deep to the ITB from anterior to posterior. (ACL, anterior cruciate ligament; ITB, iliotibial band; RF, rectus femoris; ST, semitendinosus.)

**FIGURE 10:**
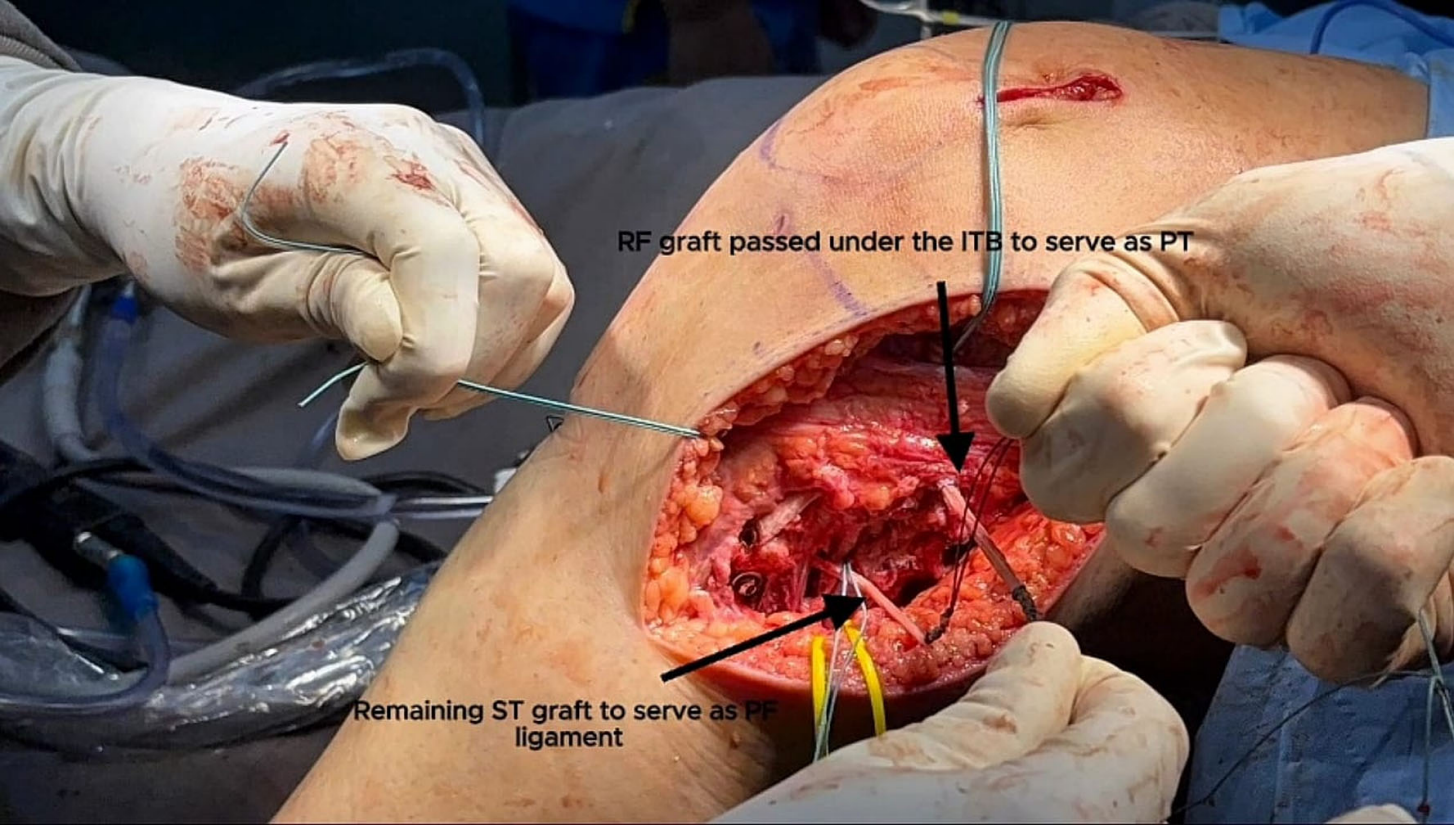
Lateral view of the left knee after passage of the remaining grafts deep to the ITB. After passage of the ST graft through the fibular tunnel to serve as the FCL, both the ST and RF grafts are then passed through the PLC tibial tunnel. Consequently, the ST graft serves as the PFL, whereas the RF graft serves as the PT. (FCL, fibular collateral ligament; ITB, iliotibial band; PF, popliteofibular; PFL, popliteofibular ligament; PLC, posterolateral corner; PT, popliteus tendon; RF, rectus femoris; ST, semitendinosus.)

Under arthroscopy, the ACL graft (double‐stranded RF + partial single‐stranded ST) is passed anterogradely and secured with 9 × 25 and 9 × 30 mm interference screws (Ortolux, Drenolux Comércio de Produtos Médicos Ltda., São Paulo, SP, Brazil) in the femoral and tibial tunnels, respectively (Figure 8). The remaining single‐stranded RF and ST grafts are passed deep to the iliotibial band from anterior to posterior (Figure 9). The ST graft is routed through the fibular tunnel from anterior to posterior and secured at 30° knee flexion with a 7 × 20 mm interference screw, serving as the FCL. The remaining ST and RF grafts are passed through the PLC tibial tunnel, from posterior to anterior, and fixed at 60° flexion with an 8 × 30 mm screw, restoring the PFL (ST) and PT (RF) (Figure 10). A physical examination is carried out to check the stability of the knee after the reconstruction.

Table 2 lists the pearls and pitfalls of this surgical technique.

**TABLE 2:** Pearls and Pitfalls of the Combined Anterior Cruciate Ligament and Posterolateral Corner Reconstruction With a Single Femoral Tunnel Using Rectus Femoris and Semitendinosus Autografts

**Surgical Steps**	**Pearls**	**Pitfalls**
Harvesting of the RF graft	‐ A 3‐cm incision in the suprapatelar region between its middle and lateral thirds, with identification of both RF and vastus intermedius planes ‐ Harvesting is done with the knee flexed at 30°	‐ Incision should be made more laterally to avoid the vastus medialis ‐ Separate the superficial and deep layers of the rectus femoris before graft harvesting to prevent intra‐articular penetration
Tunnel drilling	‐ Drill the single femoral tunnel, which should be positioned proximal and posterior to the lateral epicondyle	‐ A very anterior/vertical femoral tunnel trajectory increases the risk of convergence and tunnel widening
RF graft preparation	‐ The rectus femoris graft is divided into 2 parts, and the portion intended for the ACL should be prepared first	‐ The graft diameter and length must be assessed before creating the tunnels to avoid incompatibilities and to ensure that the PLC reconstruction is not compromised
Graft passage and fixation	‐ Fixation of the ACL graft first with the knee flexed at 90°, followed by the fixation of the PLC graft with the knee at 30° flexion, neutral rotation, and slight valgus	‐ Overtensioning the PLC in extension increases the risk of limited range of motion and postoperative stiffness
Intraoperative examination	‐ Assessment of patellar tracking and performing an arthroscopic inspection for graft impingement	‐ Neglecting the intraoperative dial test increases the risk of rotational undercorrection

ACL, anterior cruciate ligament; PLC, posterolateral corner; RF, rectus femoris.

## DISCUSSION

The combined ACL‐PLC reconstruction using a single femoral tunnel is grounded in established anatomical and biomechanical principles and addresses a key challenge in multiligament surgery: tunnel convergence. Restoration of the FCL and PFL vectors is essential for controlling varus and external rotation, as shown in in vivo models correlating structural injury with reproducible instability patterns.^1^, ^2^ Additional lateral extra‐articular procedures have recently been proposed to augment rotational control in combined ACL and PLC injuries; however, their routine use remains controversial, and further clinical studies are required to determine whether they provide superior outcomes compared with anatomic ACL and PLC reconstruction alone.^12^, ^13^ Furthermore, nonanatomic PLC reconstructions have shown higher failure rates compared with anatomic techniques, supporting the reconstructive strategy adopted in this technique.^3^

The use of a single femoral tunnel shared by the ACL and PLC allows preservation of bone stock while simplifying fixation, provided that anatomic footprints and safe tunnel trajectories are respected to maintain adequate bone bridges.^4^, ^5^, ^7^, ^12^, ^14^, ^15^ Tunnel orientation is critical, particularly in the PLC, where drilling angles directly influence construct safety and biomechanical behavior. Cadaveric and imaging studies have shown safe and reproducible trajectories that minimize tunnel conflict in multiligament reconstructions.^5^ For ACL reconstruction, consistent landmarks such as the posterior cartilage of the lateral femoral condyle improve tunnel positioning accuracy even in complex cases.^6^

In this technique, a single femoral tunnel accommodates the ACL graft while providing a lateral exit for proximal PLC fixation, maintaining anatomic reconstruction and reducing the risk of tunnel convergence.

The described graft configuration optimizes graft utilization and biomechanical function. The ACL is reconstructed using a combined construct of double‐bundle RF and single‐bundle ST, leveraging the predictable morphology of the RF and the handling characteristics of the ST. The remaining single portions of both grafts are then used for PLC reconstruction, restoring the FCL and PFL in accordance with anatomic principles and previously described techniques.^7^, ^8^, ^9^, ^10^ When necessary, the peroneus longus tendon remains a viable alternative with outcomes comparable to hamstring grafts in ACL reconstruction, allowing flexibility in graft selection without reliance on allografts.^10^, ^11^ Adequate graft preparation and fixation remain essential, and techniques that enhance fixation strength, such as the modified finger‐trap sutures, may further optimize construct stability.^16^

Previous studies have shown reliable outcomes with anatomic PLC reconstruction using autografts and various tunnel configurations.^7^, ^8^, ^9^, ^10^ Additionally, combined ACL‐PLC reconstruction with a single femoral tunnel has been described, supporting the feasibility of shared femoral fixation.^4^ The distinguishing feature of the present technique lies in its graft configuration, which maximizes autograft use while maintaining appropriate graft length and enabling sequential tensioning. The described fixation sequence—beginning with the ACL, followed by distal PLC, and concluding with proximal PLC fixation—facilitates controlled graft tensioning under physiologic conditions. Both biomechanical and clinical meta‐analyses have shown no significant differences between fibular‐based and tibiofibular‐based PLC reconstructions in restoring varus and rotational stability, suggesting comparable effectiveness of both constructs, and the choice should be individualized based on surgical preference and patient anatomy.^17^, ^18^

This technique reduces the risk of tunnel convergence, preserves bone stock with a single femoral tunnel, and uses predictable autografts from 1 limb, avoiding allografts. Limitations include technical demands, variability in RF morphology, and the need for careful tunnel planning.^4^, ^5^, ^6^ Further studies are required to evaluate clinical outcomes. Overall, it provides a reproducible and anatomically consistent option for combined ACL‐PLC reconstruction while maintaining biomechanical integrity. Table 3 shows the advantages and disadvantages of the described technique.

**TABLE 3:** Advantages and Disadvantages of Combined Anterior Cruciate Ligament and Posterolateral Corner Reconstruction With a Single Femoral Tunnel Using Rectus Femoris and Semitendinosus Autografts

**Advantages**	**Disadvantages**
Reduction in surgical time compared with techniques that use multiple femoral tunnels	Limited clinical experience and a lack of long‐term follow‐up studies involving the use of the rectus femoris tendon in multiligament reconstructions
Lower procedural costs because of reduced need for implants and disposable materials	Requires specialized training for harvesting and preparing the rectus femoris graft
Anatomic reproduction of ligament insertions with a lower risk of femoral tunnel convergence	Potential donor‐site morbidity from the rectus femoris harvest, with possible temporary reduction in quadriceps strength
Preservation of the gracilis tendon, helping maintain hamstring function	
Elimination of the need for allografts, reducing the risk of cross‐contamination, disease transmission, and immune reactions	
Use of the rectus femoris tendon, which generally provides greater length and diameter compared with other graft options, facilitating combined reconstruction	

## DECLARATION OF GENERATIVE AI AND AI‐ASSISTED TECHNOLOGIES IN THE WRITING PROCESS

During the preparation of this work, the authors used ChatGPT (OpenAI, GPT‐5.3) to assist with language editing. After using this tool, the authors reviewed and edited the content as needed and took full responsibility for the content of the publication.

## DISCLOSURES

The authors (P.H.O.P., R.G.G., F.F.S., S.E.O., R.S.A.) declare that they have no known competing financial interests or personal relationships that could have appeared to influence the work reported in this article.
